# drawProteins: a Bioconductor/R package for reproducible and programmatic generation of protein schematics

**DOI:** 10.12688/f1000research.14541.1

**Published:** 2018-07-18

**Authors:** Paul Brennan

**Affiliations:** 1 Centre for Medical Education, School of Medicine, Cardiff University, Cardiff, Wales, UK

**Keywords:** protein, schematic, BIOCONDUCTOR, R package, visualization.

## Abstract

Protein schematics are valuable for research, teaching and knowledge communication. However, the tools used to automate the process are challenging. The purpose of the drawProteins package is to enable the generation of schematics of proteins in an automated fashion that can integrate with the Bioconductor/R suite of tools for bioinformatics and statistical analysis. Using UniProt accession numbers, the package uses the UniProt API to get the features of the protein from the UniProt database. The features are assembled into a data frame and visualized using adaptations of the ggplot2 package. Visualizations can be customised in many ways including adding additional protein features information from other data frames, altering colors and protein names and adding extra layers using other ggplot2 functions. This can be completed within a script that makes the workflow reproducible and sharable.

## Introduction

Protein schematics are abundant in research papers, reviews, text books and on the internet ^1^. Thus, they represent one of the most common molecular visualizations shown and seen by researchers and students. Constructing protein schematics is often time consuming and is not performed in a reproducible and easily shared manner. The schematics frequently reflect domain expertise, but often also reflect the opinions of an individual researcher in a manner that is not obvious.

There are solutions in other languages: a Java and JavaScript tool ^1, 2^ that can be used for protein visualization. For visualization on the internet, there is also the BioJS solution, which can be used for proteins ^3^. Both of these tools are useful but not easily integrated into the Bioconductor workflow. The GenVisR package contains the option to produce highly customisable publication-quality graphics for genomic data ^4^. The focus on genomic data reduces the usefulness of drawing protein schematics, particularly those illustrating multiple proteins and protein families.

For these reasons, a protein visualization package was produced using R to allow compatibility with the Bio- conductor suite of bioinformatics packages. It uses the UniProt Proteins API ^5, 6^ as a resource of protein features and the ggplot2 package ^7^ as a basis for drawing the schematics. Multiple proteins can be drawn from similar or different families. The only limitation is the availability of UniProt entries.

Schematic customisation is possible. Protein chains, domains, regions, motifs or phosphorylation sites can be drawn separately or together. Colors can be altered and protein names (labels) can be changed. All of this can be done in a scripted manner that facilitates code sharing, visualization reproducibility and good practice in scientific computing ^8^.

## Methods

### System requirements

The Bioconductor pacakge, drawProteins, is designed to work with Bioconductor 3.7 and R version 3.5.

### Implementation

This package has been created to allow the creation of protein schematics based on the data obtained from the Uniprot Protein Database.

The basic workflow of drawProteins is:

1. To provide one or more Uniprot IDs
2. Get a list of feature from the Uniprot API
3. Draw the chains of the proteins
4. Add features as desired

drawProteins uses the package httr to interact with the Uniprot API and extract a JSON object into R. The JSON object is used to create a data frame. Adaptations of the graphing package ggplot2 are then used to create the protein schematic.

### Operation

***Getting the data from Uniprot.*** Currently, drawProteins obtains the protein feature information from the UniProt Protein API ^5, 6^. At least one working Uniprot accession number must be provided. More than one can be provided but they must be in the same vector, separated by a space. The space is replaced to create a url that can be used to query the Uniprot API ^9^.

The get_features() function uses the Uniprot API to return the features of a protein - the chain, domain information and other annotated features such as “repeats” and “motifs”. Post-translational modifications, such as phosphorylations, are also provided by the UniProt API.

The httr::content() function is then used to extract the content. From the get_features() function, this will provide lists of lists. The length of the parent lists corresponds to the number of accession numbers provided. Interestingly, the order is different to that of the UniProt accession numbers provided. The lists inside the parent list are a list of six, one for each protein, that contains names of the proteins and the features.

As an example, the script below will retrieve, from UniProt, the details of a the human version of a protein called RelA or NF-kappaB, p65, a well-studied transcription factor ^10^.

With internet access, this can be retrieved from Uniprot with this code:

library(drawProteins)
library(ggplot2)
# UniProt accession number for human rel A is Q04206
rel_json <- drawProteins::get_features("Q04206")

## [1] "Download has worked"

***Turning Uniprot data into a dataframe.*** The next step in the workflow is to convert the data from the Uniprot API into a dataframe that can be used with ggplot2.

The feature_to_dataframe() function will convert the list of lists of six provided by the get_features() function to a dataframe, which can then be used to plot the schematics.

The feature_to_dataframe() function will also add an “order” value to allow plotting. The order goes from the bottom in the manner of a graph.

rel_data <- drawProteins::feature_to_dataframe(rel_json)

The rel_data object is a data frame with 9 variables and observations that include protein features. The variables are show below. A data frame of this type could be created independently of UniProt.

str(rel_data)

## ’data.frame’:    69 obs. of 9 variables:
##  $ type       : chr  "CHAIN" "DOMAIN" "REGION" "MOTIF" ...
##  $ description: chr  "Transcription factor p65" "RHD" "Activation domain" "Nuclear localization signal" 
##  $ begin      : num  1 19 415 301 536 1 38 38 122 123 ...
##  $ end        : num  551 306 459 304 544 1 38 38 122 123 ...
##  $ length     : num  550 287 44 3 8 0 0 0 0 0 ...
##  $ accession  : chr  "Q04206" "Q04206" "Q04206" "Q04206" ...
##  $ entryName  : chr  "TF65_HUMAN" "TF65_HUMAN" "TF65_HUMAN" "TF65_HUMAN" ...
##  $ taxid      : int  9606 9606 9606 9606 9606 9606 9606 9606 9606 9606 ...
##  $ order      : int  1 1 1 1 1 1 1 1 1 1 ...

***Draw the canvas, protein chains and domains.*** The first step is to create the plot area with the draw_canvas() function. The x-axis of the canvas is based on the length of the protein (or the longest protein in the case of drawing multiple proteins). The y-axis is based on the number of proteins being drawn. The draw_canvas() function requires a data frame.

Usually, the next step is to draw the protein chains using the draw_chains() function. This requires a ggplot2 object and a data frame in that order. The data frame does not have to be the same as that used for draw_canvas() but must contain the variables type, description, begin, end, entryName, order.

**Figure 1.**
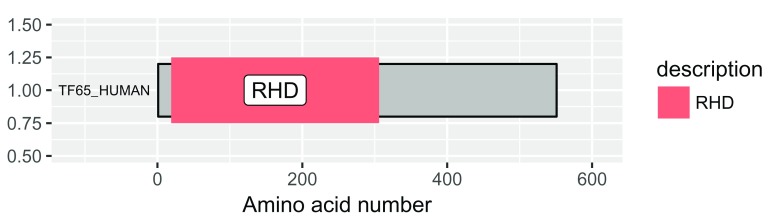
Protein domain schematic of RelA/p65. The default output gives a grey background and labels the domain. RHD = Rel Homology Domain.

Protein domains can be added with the draw_domains() function, which also requires a ggplot2 object and a data frame in that order. Again, the data frame does not have to be the same as that obtained from UniProt but must contain the variables type, description, begin, end, and order. Thus custom domains can be added with the draw_domains() function. Note that the chain and the domain are drawn to scale in terms of their number of amino acids ( Figure 1).

p <- draw_canvas(rel_data)
p <- draw_chains(p, rel_data, label_size = 2.5)
p <- draw_domains(p, rel_data)
p

To show this visualization better, a white background helps, as does removing the y-axis and the grid ( Figure 2). Changing the size of the text using the base_size argument also aids visualization. This can be done with this code:

# white background and remove y-axis
p <- p + theme_bw(base_size = 10) + # white background
    theme(panel.grid.minor=element_blank(),
        panel.grid.major=element_blank()) +
    theme(axis.ticks = element_blank(),
        axis.text.y = element_blank()) +
    theme(panel.border = element_blank())
p

**Figure 2.**
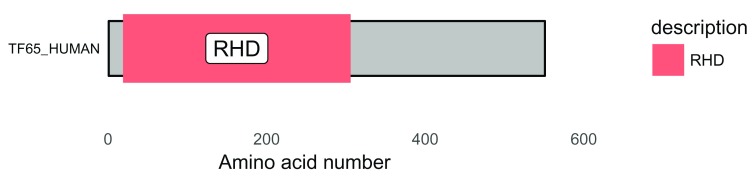
Protein domain schematic of RelA/p65. The background can be customized using theme functions from ggplot2. RHD = Rel Homology Domain.

## Use cases

### Putting the RelA workflow together and adding titles

The UniProt API provides information on protein regions, protein motifs and protein phosphorylation sites. By using the functions shown in the script below, it is possible to show the features of the protein desired to create. Altering colors and adding customisation is possible.

**Figure 3.**
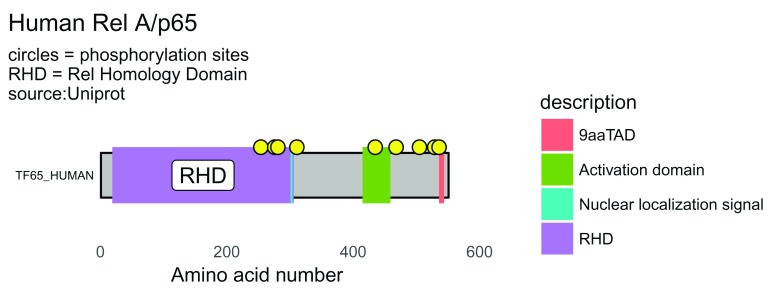
More detailed protein domain schematic of RelA/p65. By drawing the domains, regions and motifs a more detailed protein schematic is generated. RHD = Rel Homology Domain; TAD = Transactivation Domain. Yellow circles denote phosphorylation sites.

For the human protein RelA, also known as the p65 subunit of NFkappaB, a transcription factor with diverse functions including a role in leukaemia, inflammation and cancer, here is a good workflow that generates a nice schematic of the protein showing domains and phosphorylation sites ( Figure 3).

draw_canvas(rel_data) -> p
p <- draw_chains(p, rel_data, label_size = 2)
p <- draw_domains(p, rel_data)
p <- draw_regions(p, rel_data) # add regions
p <- draw_motif(p, rel_data)   # add motifs
p <- draw_phospho(p, rel_data, size = 3) # add phosphorylation sites

p <- p + theme_bw(base_size = 10) + # white backgnd & change text size
    theme(panel.grid.minor=element_blank(),
        panel.grid.major=element_blank()) +
    theme(axis.ticks = element_blank(),
        axis.text.y = element_blank()) +
    theme(panel.border = element_blank())

# add titles
rel_subtitle <- paste0("circles = phosphorylation sites\n",
                "RHD = Rel Homology Domain\nsource:Uniprot")

p <- p + labs(title = "Human Rel A/p65",
                subtitle = rel_subtitle)
p



### Drawing schematic for multiple proteins

**Figure 4.**
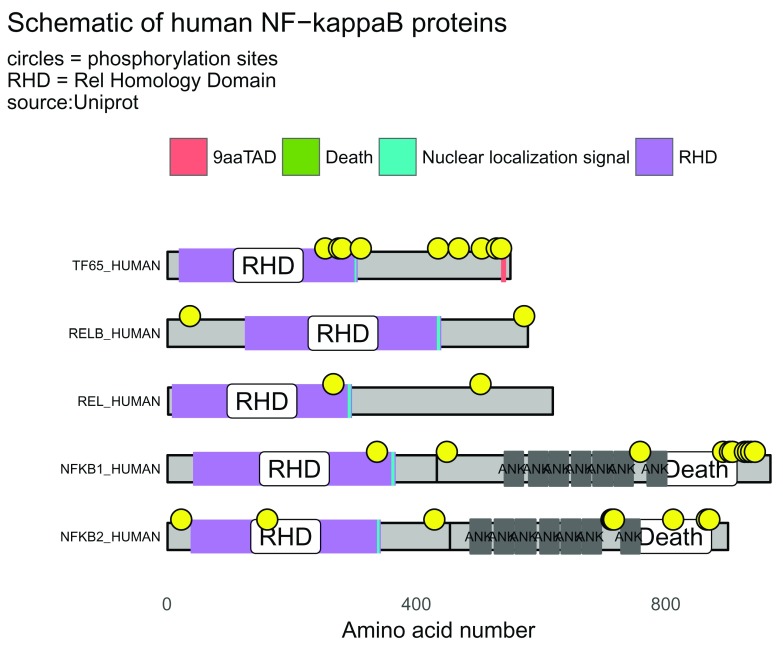
Protein domain schematic of human NF-kappaB proteins. The five members of the NF-kappaB transcription factors family can be illustrated by drawing the domains, regions and motifs as detailed on the UniProt database. The lengths of the chains, domains and motifs are proportional to the number of amino acids. RHD = Rel Homology Domain; TAD = Transactivation Domain. Yellow circles denote phosphorylation sites.

With internet access, the script below shows the workflow and generates a visualization of the five human proteins of the NF-kappaB transcription factor family ( Figure 4).

# accession numbers of five NF-kappaB proteins
prot_data <- drawProteins::get_features("Q04206 Q01201 Q04864 P19838 Q00653")

## [1] "Download has worked"

prot_data <- drawProteins::feature_to_dataframe(prot_data)

p <- draw_canvas(prot_data)
p <- draw_chains(p, prot_data, label_size = 2)
p <- draw_domains(p, prot_data)
p <- draw_repeat(p, prot_data)
p <- draw_motif(p, prot_data)
p <- draw_phospho(p, prot_data, size = 4)

# background and y-axis
p <- p + theme_bw(base_size = 10) + # white backgnd & change text size
    theme(panel.grid.minor=element_blank(),
         panel.grid.major=element_blank()) +
    theme(axis.ticks = element_blank(),
         axis.text.y = element_blank()) +
    theme(panel.border = element_blank())

# add titles
rel_subtitle <- paste0("circles = phosphorylation sites\n",
                  "RHD = Rel Homology Domain\nsource:Uniprot")

p <- p + labs(title = "Schematic of human NF-kappaB proteins",
                  subtitle = rel_subtitle)

# move legend to top
p <- p + theme(legend.position="top") + labs(fill="")
p

The proteins, domains and phosphorylation sites (yellow circles) are drawn and positioned according to amino acid number.

### Working with BioMart

**Figure 5.**
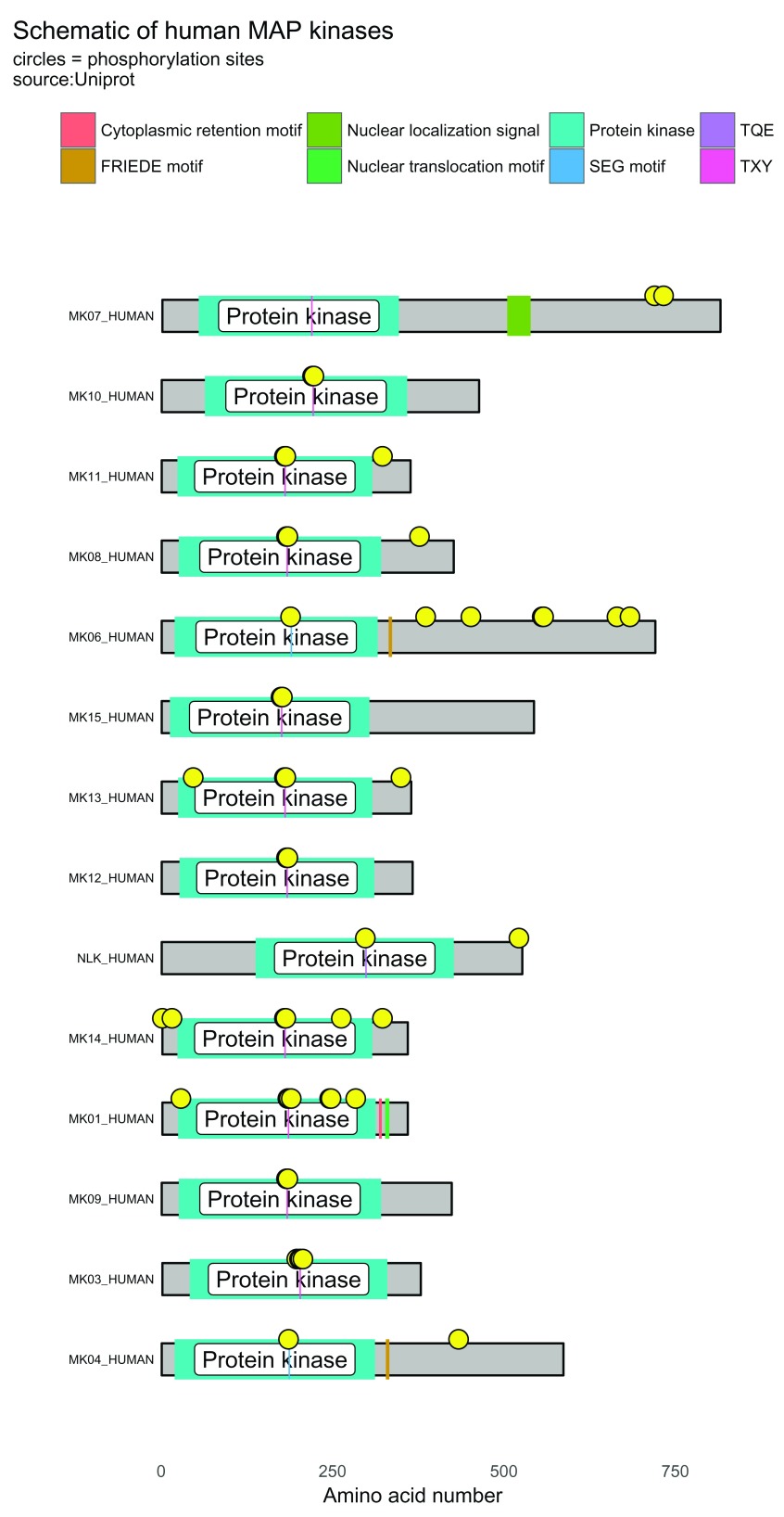
Protein domain schematic of human MAP kinases. Using bioMart with the Gene Ontology term for "MAP kinase activity", it is possible to draw multiple human MAP kinases using data from UniProt. Yellow circles denote phosphorylation sites.

It is possible to use bioMart ^11, 12^ to pull out the UniProt accession numbers for a Gene Ontology (GO) term. For example, the GO term for “MAP kinase activity”. This has a GO number of GO:0004707. This example script borrows heavily on the biomaRt users guide written by Steffen Durinck, Wolfgang Huber and Mike Smith ^13^. The script below generates a visualization containing 14 protein schematics ( Figure 5).

# install bioMart if you haven’t used it before
# remove the hash tags...
# source("http://www.bioconductor.org/biocLite.R ")
# biocLite()
# biocLite("biomaRt")
library(biomaRt)
# chosing a database = MART and a dataset - gets more focussed each step...
ensembl = useMart("ensembl",
                    dataset="hsapiens_gene_ensembl")

# Retrieve all entrezgene identifiers and HUGO gene symbols of genes which have
# a “MAP kinase activity” GO term associated with it.
# this is the GO:0004707
# this gives 14 proteins....
# create output in a dataframe and add uniprotswissprot
# which is the UniProt ID
output <- getBM(attributes = c(’uniprotswissprot’,
                                   ’hgnc_symbol’),
                  filters = ’go’,
                  values = ’GO:0004707’,
                  mart = ensembl)

# returns a dataframe... pull out uniprotIDs for drawing...
uniprotIDs <- output$uniprotswissprot
# get rid of blank entries - turn into NA
uniprotIDs[uniprotIDs==""] <- NA
# remove NA
uniprotIDs <- na.omit(uniprotIDs)
# make the IDs characters
uniprotIDs <- as.character(uniprotIDs)
# just the unique ones
uniprotIDs <- unique(uniprotIDs)
# combine into one element
uniprotIDs <- paste(uniprotIDs, collapse = " ")
# this can now be used in drawProteins

# now get features from Uniprot
library(magrittr)

uniprotIDs %>%
  drawProteins::get_features() %>%
  drawProteins::feature_to_dataframe() ->
  prot_data


## [1] "Download has worked"

# data frame with 722 observations

library(ggplot2)
# basic drawing
p <- draw_canvas(prot_data)
p <- draw_chains(p, prot_data, label_size = 2)
p <- draw_domains(p, prot_data)
p <- draw_repeat(p, prot_data)
p <- draw_motif(p, prot_data)
p <- draw_phospho(p, prot_data, size = 4)

# background and y-axis
p <- p + theme_bw(base_size = 10) + # white background and change text size
  theme(panel.grid.minor=element_blank(),
    panel.grid.major=element_blank()) +
  theme(axis.ticks = element_blank(),
    axis.text.y = element_blank()) +
  theme(panel.border = element_blank())
  
# add titles
p <- p + labs(title = "Schematic of human MAP kinases",
  subtitle = "circles = phosphorylation sites\nsource:Uniprot")
  
# move legend to top
p <- p + theme(legend.position="top") + labs(fill="")

p

### Customizing the draw functions

Various customizations are possible:

1. Alter chain color and outline.
2. Change the labels to a custom list (but remember that the plots are drawn from the bottom up)
3. Change the size and color of the phosphorylation symbols.

**Figure 6.**
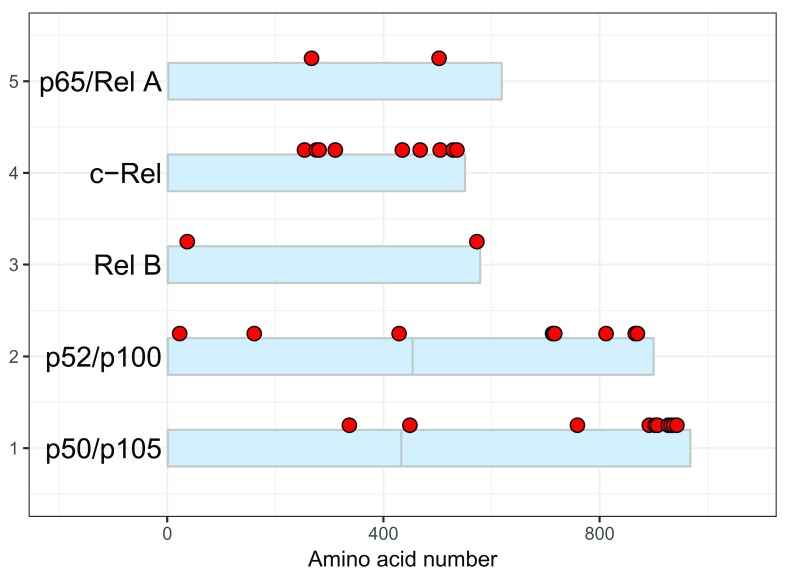
Customizing the protein schematic of the NF-kappaB family. Using arguments in the draw_chains() and draw_phospho() functions, it is possible customize colors and labels.

These are illustrated with the script below which generates Figure 6.

data("five_rel_data")
p <- draw_canvas(five_rel_data)
p <- draw_chains(p, five_rel_data,
             fill = "lightsteelblue1",   # chain fill color
             outline = "grey",            # chain outline
             labels = c("p50/p105",      # custom protein names
                          "p50/p105",
                          "p52/p100",
                          "p52/p100",
                          "Rel B",
                          "c-Rel",
                          "p65/Rel A"),
             label_size = 5)
# change size and color of phosphorylation sites
p <- draw_phospho(p, five_rel_data, size = 3, fill = "red")
p + theme_bw()

## Discussion

After 20 years of manual drawing of protein schematics and experience in proteomic studies ^14– 17^, the need for a more sustainable and programmatic method seemed important and worthwhile. It seemed wise to develop an approach that would integrate protein visualizations with other bioinformatic tools available in Bioconductor. This package represents an approach to enabling the reproducible and programmatic generation of protein schematics.

The plan is to develop this package further in terms of generating use cases and adding features. A list of issues for future development has already been added by the author on GitHub. Bug reports, feedback on desired features or code contributions can be made through GitHub.

The challenge with protein visualization is that specialist domain knowledge sometimes trumps databases. Thus, while integrating knowledge from UniProt is an excellent starting point, it is also essential to allow customisation of plots. This can be done by adding or removing information about the proteins, protein features and post-translational modification to the dataframe object made with R.

## Data availability

All data underlying the results are available as part of the article and no additional source data are required.

## Software availability

**The drawProteins package is available at:** http://bioconductor.org/packages/drawProteins/

**Source code is available at:** https://github.com/brennanpincardiff/drawProteins

**Archived source code as at time of publication:** https://github.com/brennanpincardiff/drawProteins/tree/v1.0.2 and https://doi.org/10.5281/zenodo.1306619 ^18^

**License:** MIT+
