# Supplementary material for: drawProteins: a Bioconductor/R package for reproducible and programmatic generation of protein schematics
Source: F1000Res. 2018 Jul 18;7:1105. [Version 1] doi: 10.12688/f1000research.14541.1 (PMC6107989; doi:10.12688/f1000research.14541.1)
Supplement: Peer review history

# Peer review: Referee response for version 1

**DOI:** 10.5256/f1000research.15828.r37075

**Published:** 2018-08-22

**Authors:** Malachi Griffith

**Affiliations:** 1 McDonnell Genome Institute, Washington University School of Medicine, St. Louis, MO, USA

The author describes the creation of a Bioconductor/R package for generic visualization of 2D protein diagrams.  The tool does not address 3D visualizations or visualization of protein sequences or alignments. Rather it allows the user to view the position of protein annotations within an arbitrary number of proteins. By default, UniProt sequence identifiers and associated annotations are supported. The demonstrated examples depict protein domains/motifs and phosphorylation sites.  Identifying a set of proteins to view from the Gene Ontology via bioMart is also demonstrated. There are likely to be many additional useful queries that could be achieved by similar bioMart queries.

 The package leans on ggplot2 functionality for the actual drawing of each protein schematic. The drawings consist of simple elements: colored rectangles and circles that can be nested, overlaid, and labeled.  A few simple use cases are illustrated.  The writing is brief and straightforward. The code is available under an open access document. 

 I was able to install the package and run through the examples in the paper in a few minutes with only minor difficulties.

 In particular, I had trouble getting this line to work in the example:

 ```

 output <- getBM(attributes = c('uniprotswissprot', 'hgnc_symbol'), filters = 'go', values = 'GO:0004707', mart = ensembl)

 ```

 Perhaps some problem with the way the single quotes are being encoded in that section?

 Overall the tool is quite basic but also appears to be customizable. I anticipate it could be useful to many who wish to automate visualization of protein drawings and integrate these with visualization of various types omics data.

I have read this submission. I believe that I have an appropriate level of expertise to confirm that it is of an acceptable scientific standard.

# Peer review: Referee response for version 1

**DOI:** 10.5256/f1000research.15828.r37074

**Published:** 2018-08-22

**Authors:** Matej Lexa

**Affiliations:** 1 Department of Machine Learning and Data Processing, Faculty of Informatics, Masaryk University, Botanicka 68a, 60200 Brno, Czech Republic

The paper describes a protein annotation visualization tool for R/Bioconductor that has already been in use for more than a year. The software is mature, I was able to install it easily, all the examples of code in the manuscript are functional. I only noticed a small deviation in the biomaRt example, most likely caused by slightly different data returned by biomaRt.

 The manuscripts describes the purpose and functioning of the package in sufficient detail. However, I found the introduction a bit too brief, especially in terms of mentioning other similar software and advantages of drawProteins. For example, I found a Bioconductor package called Pviz that may be able to generate protein annotation visualizations.

 As a minor remark, the last sentence in this paragraph on page 3 seems unclear to me:

 "The feature_to_dataframe() function will also add an “order” value to allow plotting. The order goes from the bottom in the manner of a graph."

 Finally, a suggestion. The software would be even more useful if it could handle reading and writing of standard protein annotation files in GFF or similar format. UniProt wikipedia page describes how it is applied to proteins in this example: https://www.uniprot.org/uniprot/P0A7B8.gff Although GFF is much more popular with genomic data, it seems to be ocassionally used for annotating protein sequences.

I have read this submission. I believe that I have an appropriate level of expertise to confirm that it is of an acceptable scientific standard.
